# Molecular Regulation of Growth in Aquaculture: From Genes to Sustainable Production

**DOI:** 10.3390/life15121831

**Published:** 2025-11-28

**Authors:** Dana Andreea Șerban, Cristian-Alin Barbacariu, Mihaela Ivancia, Șteofil Creangă

**Affiliations:** 1 Research and Development Station for Aquaculture and Aquatic Ecology, Alexandru Ioan Cuza University Iasi, 11, Carol I Blvd., 700506 Iași, Romania; dana.serban@uaic.ro; 2 Faculty of Food and Animal Sciences, University of Life Sciences, Ion Ionescu de la Brad Iaşi, Mihail Sadoveanu Alley 6-8, 700490 Iași, Romania; mihaela.ivancia@iuls.ro (M.I.); steofil.creanga@iuls.ro (Ș.C.)

**Keywords:** aquaculture genetics, polyploid genomics, growth regulation, genomic selection, CRISPR-Cas9, epigenetics, climate adaptation

## Abstract

The global aquaculture industry produces 91 million tons annually, yet achieving sustainable growth optimization remains constrained by incomplete understanding of regulatory system integration, polyploid genomic complexity, and disconnected molecular-environmental approaches. This systematic review synthesizes 180 peer-reviewed articles (1992–2025) from four databases, revealing that growth regulation operates through integrated multi-level networks: the GH-IGF axis, TGF-β/myostatin signaling, and epigenetic mechanisms responding dynamically to environmental inputs. Research acceleration is evident, with 52.2% of studies published during 2020–2025. Whole-genome duplication events created expanded gene repertoires enabling sophisticated regulatory control while presenting breeding challenges in polyploid species. CRISPR-Cas9 myostatin knockout achieves 15–30% growth enhancement, though practical implementation faces regulatory and economic barriers. DNA methylation and microRNAs enable environmental adaptation and transgenerational trait inheritance, offering complementary approaches to conventional breeding. Climate-resilient strain development requires integrating polyploid breeding methodologies, multi-omics phenotyping platforms, and validated epigenetic markers. Sustainable aquaculture intensification through precision genetics demands coordinated infrastructure development, harmonized regulatory frameworks, and international collaboration to address food security while adapting to climate change. This synthesis establishes research priorities bridging molecular mechanisms with practical applications for sustainable production enhancement.

## 1. Introduction

The global aquaculture sector has experienced remarkable growth, generating over 91 million tons annually and serving as a cornerstone of worldwide food security strategies [1,2]. This expansion reflects increasing global protein demand and recognition of aquaculture’s potential for sustainable food production [3]. However, optimizing production efficiency through sustainable practices requires comprehensive understanding of the biological systems controlling fish development and growth.

Growth performance represents the most critical economic trait in aquaculture operations, directly determining production costs, market readiness, and profitability [4]. The industry demands enhanced growth performance and feed conversion rates to meet environmental sustainability targets [5,6]. While traditional selective breeding has proven effective, the integration of molecular biology and genomics now offers unprecedented opportunities for trait improvement at the genetic level. However, realizing this potential requires bridging fundamental molecular mechanisms with practical application in sustainable production systems.

Fish growth regulation involves intricate molecular networks integrating environmental, nutritional, and developmental signals through three primary regulatory systems. The growth hormone (GH)-insulin-like growth factor (IGF) axis functions as the central endocrine system controlling body size development [7,8], while the transforming growth factor-β (TGF-β) superfamily, particularly myostatin, acts as the primary negative regulator limiting muscle growth [9,10]. Environmental pathways modulate these core systems through temperature, nutrition, and other ecological factors, with temperature serving as the dominant modulating factor in poikilothermic fish [11,12]. Additionally, epigenetic mechanisms including DNA methylation and microRNAs provide regulatory layers that manage environmental effects on gene expression and enable transgenerational trait inheritance [13,14,15], offering opportunities for environmentally responsive breeding strategies that complement traditional genetic approaches.

Commercial aquaculture species, including common carp (*Cyprinus carpio*), goldfish (*Carassius auratus*), and various salmonid species, exhibit complex genomic architecture resulting from ancient whole-genome duplication (WGD) events [16,17]. These polyploidization events created expanded gene repertoires and enhanced regulatory mechanisms for growth control, with the teleost-specific WGD occurring 320–350 million years ago fundamentally shaping modern fish biology [16,18]. In polyploid species like common carp, these duplication events resulted in complex allotetraploid genomes containing ~50,000 genes across four sub-genomes [19], providing unique opportunities for genetic improvement through sub-functionalization, neofunctionalization, and dosage-dependent effects. However, this complexity presents substantial challenges for breeding program implementation, including intricate inheritance patterns, coordinated regulation of multiple homoeologs, and technical complications for genetic mapping and marker-assisted selection [9,20].

Advanced molecular technologies are revolutionizing aquaculture genetics through genomic selection, marker-assisted breeding, and genome editing technologies that accelerate genetic improvement while preserving genetic diversity [21,22,23]. CRISPR-Cas9 enables precise genetic modifications targeting growth performance, with myostatin knockout studies demonstrating 15–30% growth enhancements in multiple species [24,25]. However, implementation faces significant barriers including divergent global regulatory frameworks, substantial economic investment requirements, technical complexity in polyploid systems, and variable consumer acceptance across markets [26,27]. Furthermore, climate change impacts necessitate development of environmentally resilient species capable of maintaining productivity under changing conditions [28], requiring integration of thermal tolerance, hypoxia resistance, and metabolic flexibility into breeding objectives [11,29].

Despite these significant advances, three critical knowledge gaps limit our ability to optimize growth performance sustainably. First, incomplete integration of multi-level regulatory networks prevents comprehensive understanding of how GH-IGF signaling, TGF-β pathways, environmental modulation, and epigenetic control interact to determine final growth outcomes [8,12,30]. Second, limited exploitation of polyploid genomic complexity means that most breeding programs treat polyploid species using diploid-oriented approaches, failing to leverage sub-genome-specific variation, epigenetic coordination mechanisms, and heterosis contributions unique to polyploid systems [20,31,32]. Third, disconnected approaches between molecular breeding and environmental sustainability result in genetic improvement programs that optimize growth under current conditions but fail to incorporate climate adaptation, resource efficiency, and ecosystem-level sustainability as core breeding objectives [28,33,34]

This review addresses these gaps by synthesizing current understanding across core growth regulatory networks, genetic architecture and genomic applications, and sustainable integration strategies, with particular focus on polyploid species representing both the greatest complexity and opportunity in aquaculture genetics. Unlike previous reviews that examine molecular mechanisms, genomic technologies, or sustainability practices in isolation, this synthesis uniquely integrates these three perspectives to provide a comprehensive framework for sustainable molecular aquaculture development. We emphasize the special considerations required for polyploid species where complex genomic architecture creates both enhanced opportunities for genetic manipulation and substantial challenges for breeding program implementation [31,35]. By connecting precision genetics with environmental stewardship and climate adaptation, this review establishes research priorities for meeting growing global food security demands while adapting to environmental change.

The review is organized into four main sections: (1) Core Growth Regulatory Networks examines the GH-IGF axis, TGF-β/myostatin signaling, and epigenetic control mechanisms, emphasizing environmental modulation and systems-level integration; (2) Genetic Architecture and Genomic Applications explores polyploid genome complexity, comparative genomic architecture, and molecular breeding technologies including genomic selection, marker-assisted selection, and CRISPR-based genome editing; (3) Sustainable Integration and Future Directions addresses integrated multi-trophic aquaculture systems, climate adaptation strategies, emerging technologies including AI and multi-omics integration, and research priorities with implementation challenges; and (4) Conclusions synthesize key findings and outline the path forward for sustainable molecular aquaculture development.

## 2. Materials and Methods

### 2.1. Literature Search Strategy and Selection Criteria

**Figure 1:**
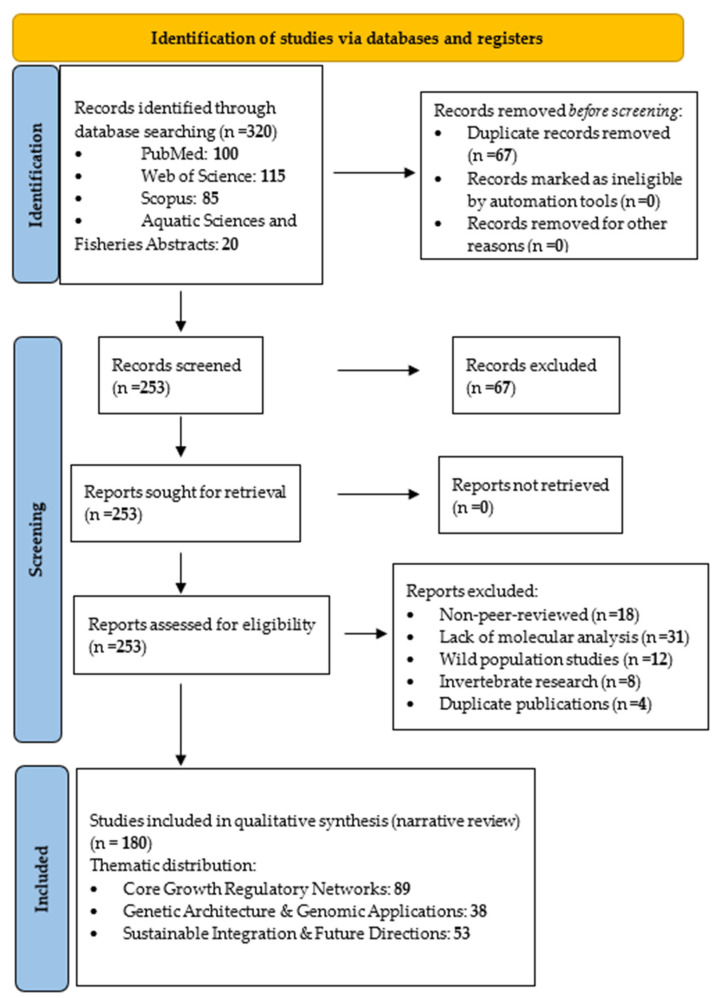
PRISMA 2020 flow diagram of the systematic literature search and study selection process [36].

A systematic literature review following PRISMA (Preferred Reporting Items for Systematic Reviews and Meta-Analyses) guidelines analyzed peer-reviewed studies on molecular growth regulation in aquaculture fish from 1992 to 2025 (Figure 1). Four major databases were searched: PubMed, Web of Science, Scopus, and Aquatic Sciences and Fisheries Abstracts.

The detailed search strategy employed Boolean operators (AND, OR), wildcards (*), and parenthetical grouping across four thematic domains:

(Set 1) “aquaculture” OR “fish farming” OR “pisciculture”

AND

(Set 2) “growth hormone” OR “GH” OR “insulin-like growth factor” OR “IGF*” OR “myostatin” OR “MSTN” OR “transforming growth factor” OR “TGF-β” OR “growth regulation”

AND

(Set 3) “gene expression” OR “genomics” OR “molecular biology” OR “genome editing” OR “CRISPR” OR “genomic selection” OR “marker-assisted selection” OR “MAS” OR “epigenetics” OR “DNA methylation” OR “microRNA” OR “miRNA”

AND

(Set 4) “teleost*” OR “cyprinid*” OR “Cyprinus carpio” OR “common carp” OR “salmonid*” OR “tilapia” OR “aquaculture species”

This strategy was supplemented with reference checking and forward citation tracking to identify additional relevant publications.

This search was supplemented with reference checking and forward citation tracking.

The screening process was conducted independently by D.A.Ș. and C.-A.B., with M.I. resolving disagreements. Inter-rater agreement was assessed using Cohen’s kappa (κ), a statistical measure quantifying agreement between raters while correcting for chance. The achieved κ = 0.87 indicates “almost perfect agreement” (κ > 0.81 threshold), demonstrating high consistency in study selection.

Inclusion criteria: English-language peer-reviewed articles investigating molecular growth regulation in aquaculture species, genetic improvement methods, genomic applications, or sustainable aquaculture practices with molecular/genetic components; original research, systematic reviews, or meta-analyses.

Exclusion criteria: From 253 screened studies, 73 were excluded: non-peer-reviewed publications (n = 18), lack of molecular/genetic analysis (n = 31), wild population focus without aquaculture relevance (n = 12), invertebrate research beyond review scope (n = 8), and duplicate publications (n = 4). This resulted in 180 studies for final analysis.

### 2.2. Study Classification and Data Extraction

**Figure 2:**
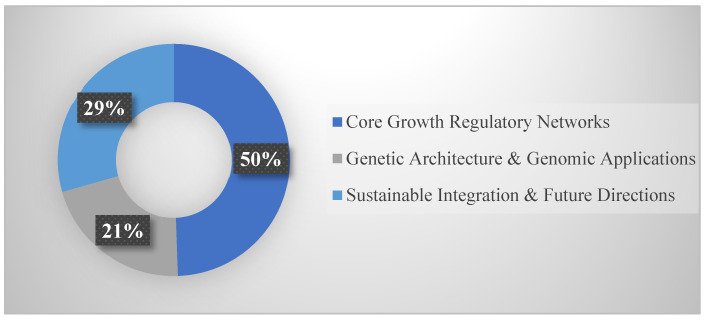
Main section distribution.

Studies were classified using a hierarchical thematic framework, with each assigned to its primary focus area (secondary themes noted but not double-counted). The 180 studies distributed across three major sections (Figure 2): Core Growth Regulatory Networks (89 studies, 49.4%), Genetic Architecture and Genomic Applications (38 studies, 21.1%), and Sustainable Integration and Future Directions (53 studies, 29.4%). Figure 2 illustrates this proportional distribution, showing that approximately half the literature addresses fundamental regulatory mechanisms, with the remainder split between applied genomic technologies and sustainability integration.

**Figure 3:**
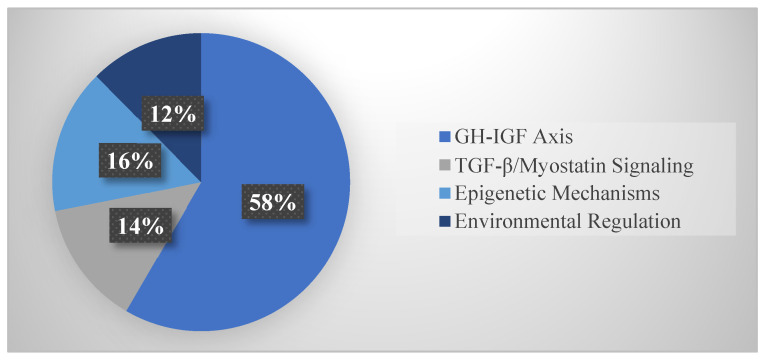
Core growth regulatory networks detailed breakdown.

Within Core growth regulatory networks (Figure 3), subsections included: GH-IGF axis (52 studies, 58.4%), TGF-β/myostatin signaling (12 studies, 13.5%), epigenetic mechanisms (14 studies, 15.7%), and environmental regulation (11 studies, 12.4%). The GH-IGF axis dominance reflects its role as the central growth regulatory pathway.

**Figure 4:**
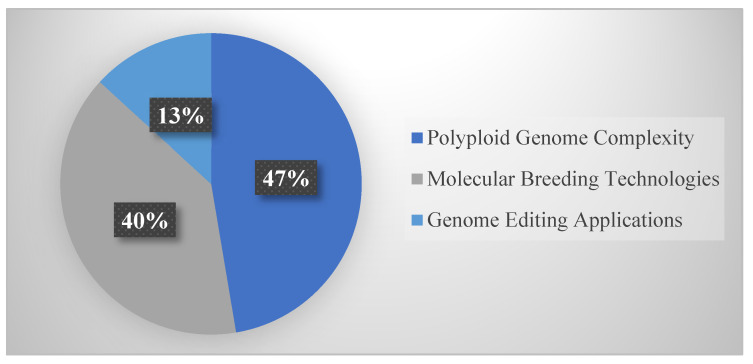
Genetic architecture & genomic applications breakdown.

Within Genetic architecture and genomic applications (Figure 4), distribution included: polyploid genome complexity (18 studies, 47.4%), molecular breeding technologies (15 studies, 39.5%), and genome editing applications (5 studies, 13.1%). The polyploid genomics emphasis reflects unique challenges and opportunities these systems present.

**Figure 5:**
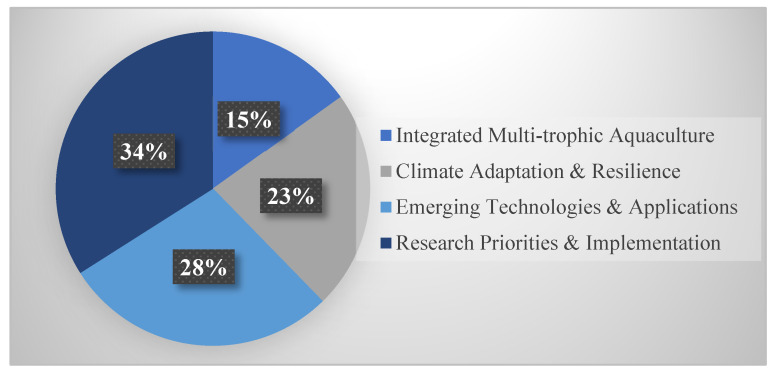
Sustainable integration & future directions breakdown.

Within Sustainable integration and future directions (Figure 5), subsections addressed: integrated multi-trophic aquaculture (8 studies, 15.1%), climate adaptation and resilience (12 studies, 22.6%), emerging technologies (15 studies, 28.3%), and research priorities and implementation (18 studies, 34.0%). Implementation challenges represent the largest subsection, indicating growing focus on translating molecular knowledge into practice.

### 2.3. Temporal and Quantitative Analysis

**Figure 6:**
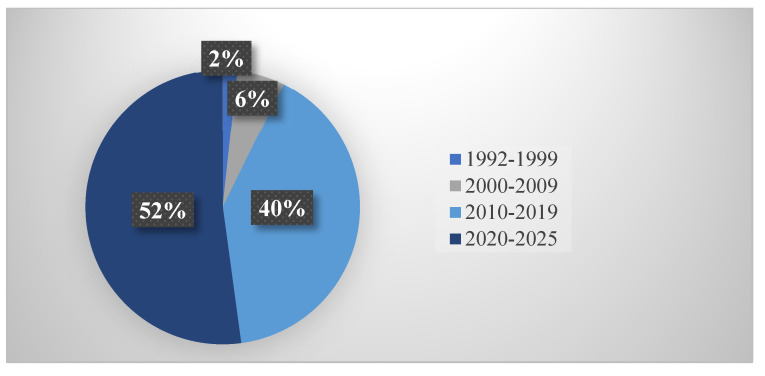
Temporal distribution analysis.

Temporal analysis revealed research acceleration, with 94 studies (52.2%) published between 2020–2025, demonstrating intensified recent activity in molecular aquaculture genetics (Figure 6). This acceleration was most pronounced in Genetic Architecture & Genomic Applications (22/38 studies, 57.9% from 2020–2025) and Core Growth Regulatory Networks (48/89 studies, 53.9% from 2020–2025).

**Figure 7:**
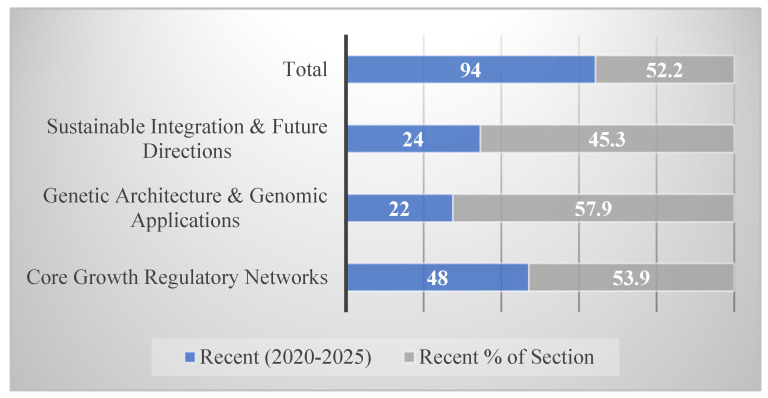
Main section distribution with recent publications.

Figure 7 displays the proportion of recent publications within each major section, with Genetic Architecture showing the highest percentage, reflecting rapid technological advances in genomics and genome editing. Figure 6 presents the complete temporal distribution across the 33-year period (1992–2025), illustrating exponential growth with particular acceleration post-2015, coinciding with CRISPR-Cas9 maturation and next-generation sequencing cost reductions.

**Figure 8:**
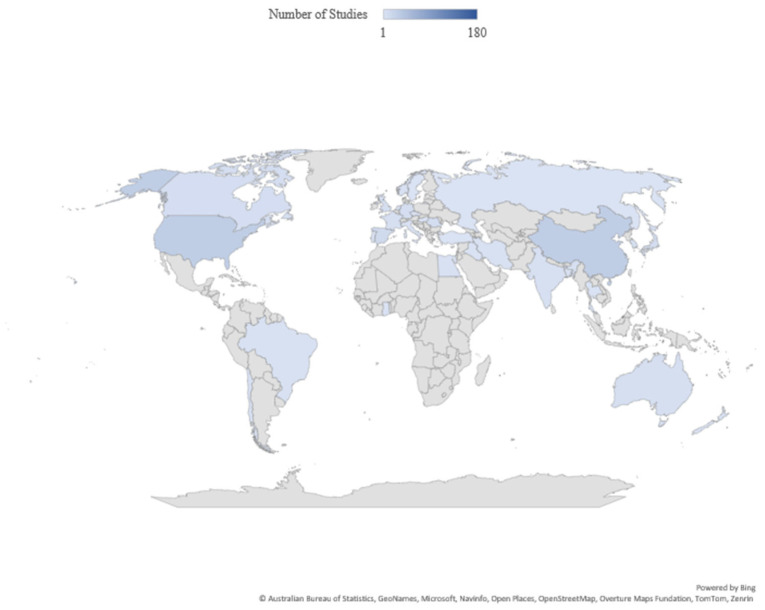
Geographic distribution of research showing regional contributions to molecular aquaculture genetics research.

Research distribution across geographic regions (Figure 8) reflects aquaculture industry maturity and molecular genetics research infrastructure investment. Based on author affiliations and study locations, major contributing regions included: East Asia (China, Japan, Republic of Korea; 42% of studies), driven by intensive aquaculture production and government research investment; Europe (EU, Norway, UK; 28%), with advanced genomic infrastructure and sustainability requirements; North America (USA, Canada; 18%), emphasizing salmonids; South Asia (India, Bangladesh, Thailand; 8%), rapidly growing with carp and tilapia focus; and Other regions (South America, Middle East, Africa, Oceania; 4%). Figure 8 visualizes this distribution, demonstrating global research activity while highlighting regional specializations.

The review emphasized common carp (*Cyprinus carpio*) due to its economic importance, well-characterized polyploid genome serving as a model system, and extensive genetic resources, with secondary focus on salmonids and tilapia representing major global taxa.

This review employs narrative synthesis rather than quantitative meta-analysis due to substantial methodological heterogeneity: diverse experimental designs (laboratory studies, field trials, population surveys, computational analyses), multiple target species with divergent physiologies, various molecular pathways with non-comparable endpoints, and diverse outcome measurements (gene expression, phenotypic traits, genomic variants, environmental responses). This heterogeneity precludes meaningful statistical pooling, making qualitative synthesis appropriate.

From each study, we extracted: target species and life stage, molecular targets (genes, pathways, regulatory mechanisms), methodological approaches, key findings and effect sizes, practical applications, and environmental contexts. These data were analyzed to identify common patterns, knowledge gaps, emerging research opportunities, and translation pathways from mechanisms to applications.

Special attention was directed to polyploid species, whose complex genomic architecture from whole-genome duplication events creates both opportunities (enhanced diversity, sub-functionalization, neofunctionalization) and challenges (complex inheritance, coordinated homoeolog regulation, technical complications). This required dedicated analysis to extract practical breeding implications from fundamental genomic research.

## 3. Core Growth of Regulatory Networks

### 3.1. GH-IGF Axis and Environmental Modulation

#### 3.1.1. Growth Hormone (*GH*) Gene Family

The *GH* gene family in fish exhibits extensive evolutionary diversity due to teleost-specific whole-genome duplications that produced numerous *GH* gene copies, enabling sub-functionalization and neofunctionalization [37,38]. The teleost whole-genome duplication event triggered extensive evolutionary changes through subsequent rediploidization processes [16,17], creating complex genomic architecture that enables sophisticated regulation of skeletal muscle growth networks [18].

Table 1 summarizes the key growth regulatory genes identified in aquaculture species, demonstrating the functional diversity within the GH-IGF axis and related pathways. These genes show distinct but coordinated roles, with GH functioning as the central regulator, IGF isoforms mediating tissue-specific effects, and myostatin providing negative feedback control to prevent excessive muscle growth. This multi-level regulatory architecture enables precise growth control under diverse environmental and nutritional conditions.

In common carp, *GH* gene polymorphisms significantly affect growth performance, particularly during critical developmental stages [39,40]. Genetic variations influence growth outcomes across different populations [41], reflecting complex polygenic control systems [42]. The 79 bp insertion/deletion polymorphism in the *IGF-I* gene 3′-flanking region associates with growth-related traits [43], while growth hormone receptor gene mutations affect productive traits across varying environmental conditions [44]. Growth hormone gene polymorphisms also influence growth characteristics in inland saline production systems [45], demonstrating the environmental context-dependency of genetic effects.

The IGF system comprises multiple components including IGF-I, IGF-II, fish-specific IGF-III, binding proteins (IGFBPs), and receptors that mediate GH’s anabolic effects [8,46]. IGF-I mRNA expression responds dynamically to dietary protein intake and temperature variations [47], while IGFBP-2 and IGFBP-3 exhibit distinct expression patterns with hepatocyte-specific hormonal control [48,49]. Fish-specific IGF-III regulates both gonadal development and somatic growth [50], with knockdown experiments revealing complex interactions between long noncoding RNAs and mRNA expression patterns [51].

**Table 1:** Key growth regulatory genes and their functions in aquaculture fish.

Gene/Gene Family	Primary Function	Regulatory Role	Aquaculture Application	Key References
GH	Central growth regulator	Controls body size development, metabolic processes	Transgenic enhancement, marker-assisted selection	[7,40]
IGF-I	Mediates GH anabolic effects	Tissue-specific growth promotion, protein synthesis	QTL mapping, expression markers	[43,47]
IGF-II	Early development regulator	Embryonic and larval growth	Developmental stage optimization	[52]
IGF-III	Fish-specific growth factor	Gonadal development, reproductive growth balance	Breeding program timing	[50]
MSTN	Negative muscle growth regulator	Limits muscle cell proliferation and differentiation	CRISPR knockout targets, selective breeding	[53,54]
IGFBP-2/3	IGF bioavailability control	Modulates IGF signaling duration and intensity	Metabolic efficiency markers	[48,49]
GHR	GH signal transduction	Environmental sensitivity modulation	Environmental adaptation breeding	[44]
TGF-β1	Muscle development coordination	Multi-tissue growth integration	System-wide growth optimization	[55]
SMAD4	TGF-β signal transduction	Transcriptional control of growth genes	Regulatory pathway targeting	[56]

GH-growth hormone; IGF-I—insulin-like growth factor-I; MSTN—myostatin; GHR—growth hormone receptor.

Molecular characterization of insulin-like growth factor receptor (IGF-1Ra) through genetic analysis and molecular docking studies provides detailed structural insights [57]. Expression studies of the GH/IGF axis in polyploid fish reveal enhanced regulatory complexity [58,59]. Understanding how various factors modulate GH and *IGF-I* gene expression benefits aquaculture applications [60], with peripheral and central regulation mechanisms showing vertebrate conservation patterns [61,62].

Functional studies including expression, purification, and biological activity assessment of recombinant carp growth hormone provide essential mechanistic insights [63]. Real-time PCR analyses demonstrate differential regulation of common carp *IGF-I* and *IGF-II* mRNA levels following GH stimulation [64], while complete molecular characterization of IGF-II through PCR-cloning and expression studies establishes foundational knowledge for growth regulation research [52].

#### 3.1.2. Environmental Temperature Effects and Stress Integration

Temperature serves as the primary environmental modulator of growth in poikilothermic fish, directly influencing gene expression, protein function, and cellular metabolism [11]. Cellular stress response mechanisms and temperature regulation systems are evolutionarily conserved across vertebrates [65]. Thermal stress triggers coordinated responses across multiple regulatory pathways, with genetic studies revealing how temperature sensing mechanisms integrate with endocrine control systems during both acute and chronic thermal exposures [29]. Elevated acclimation temperatures suppress growth through downregulation of *GH* and *IGF* gene expression while simultaneously activating stress response pathways that interfere with normal growth hormone signaling [66].

Environmental temperature during development plays critical roles in tissue histology, biochemical indicators, and GH/IGF system gene transcription [67]. The IGF-1 system follows circadian patterns that coordinate growth regulation with natural photoperiod and feeding cycles [68]. Low temperatures affect both endocrine and circadian systems, demonstrating integrated temporal control of growth processes [69]. Reproductive biocomplexity management requires sustainable approaches that account for climate change impacts [28], with environmental hypoxia functioning as an additional climate-related stressor affecting development through interactions with thermal stress and oxygen availability [70].

Environmental stressors influence growth through coordinated biological systems. Epigenetic processes mediate environmental effects on gonadal development, enabling adaptive responses [71]. Environmental factors shape gonadal miRNome, transcriptome, and metabolome through intricate molecular pathways [72]. Environmental enrichment provides fundamental approaches for improving aquaculture production systems [73], with brain-mediated integration of environmental stressors and growth regulation occurring through complex neuroendocrine networks [12,74]. MicroRNAs mediate environmental stress responses and coordinate adaptive physiological adjustments through epigenetic regulatory mechanisms [75].

#### 3.1.3. Nutritional Regulation and Metabolic Integration

Nutritional regulation involves complex molecular systems integrating nutritional signals with environmental factors and metabolic requirements [76]. Nutrient availability influences growth performance through coordinated effects on feeding behavior and metabolic processes [12]. Environmental factors interact with dietary protein levels to modulate IGF-I mRNA expression and downstream growth regulatory pathways [47].

Feed intake regulation depends on appetite-controlling neuropeptides, with apelin coordinating energy utilization, appetite, and growth through hypothalamic signaling networks [77]. Ghrelin functions as a conserved appetite regulator showing tissue-specific distribution patterns that modulate feeding behavior according to metabolic status [78,79]. Gonadotropin-releasing hormone affects growth hormone secretion, revealing hormonal integration mechanisms [80], while GnRH analogs and sex steroids demonstrate intricate endocrine relationships [81,82].

Alternative protein sources offer sustainable aquaculture solutions while preserving growth hormone and *IGF-1* gene expression, as demonstrated with detoxified Jatropha curcas kernel meal [83]. Black Soldier Fly larvae meal promotes growth and immunity through favorable nutrient–gene interactions [84]. Dietary fat source affects both metabolic pathways and growth regulatory systems [85], while plant-derived essential oils from bitter orange benefit growth performance, histology, and gene expression in common carp juveniles [86].

Short-term intermittent fasting affects growth performance, fatty acid profiles, and gene expression in European seabass [87], while starvation and refeeding cycles influence growth, immune response, and intestinal microbiota in Nile tilapia through integrated physiological mechanisms [88]. Alternative feed ingredients including grape pomace and sorghum demonstrate beneficial effects on growth performance, biochemical parameters, meat quality, gut microbiota composition, and oxidative status in common carp [89,90,91].

Probiotic and prebiotic supplementation modulates growth through multiple integrated mechanisms. *Lactobacillus delbrueckii* and *Lactococcus lactis* strains enhance growth performance through coordinated improvements in immune function, disease resistance, and antioxidant capacity [92,93]. Postbiotic compounds extend these benefits to skin mucus immunity, hepatic function, and gut microbiota structure [94]., while prebiotics specifically upregulate immune-related gene expression [95,96]. Specialized formulations such as selenium-enriched *Bacillus subtilis* additionally provide protective effects against environmental toxins [97], demonstrating that nutritional interventions can simultaneously optimize growth and stress resilience through gut microbiome modulation.

#### 3.1.4. Multi-Omics and Systems Biology Integration

Multi-omics approaches identify molecular factors affecting fish muscle quality under various aquaculture management systems through integrated genomic, transcriptomic, proteomic, and metabolomic analyses [98]. Transcriptomic profiling reveals regulatory genes and molecular pathways optimizing feed efficiency [99], while branched-chain amino acid roles in muscle growth regulation emerge from combined transcriptome and microRNA sequencing [100].

Intestinal microbial community interactions with transcriptome profiles demonstrate host–microbiome integration [101]. Comparative microRNA expression profiles during embryonic development reveal developmental regulatory mechanisms [102], while sex-specific analyses of miRNAs, lncRNAs, and circRNAs between ovaries and testes identify unique regulatory systems [103].

Transcriptomic profiling identifies tissue-specific GH/IGF system gene expression patterns and hypoxia responses [104]. Integrated transcriptome and miRNA sequencing reveals that hypoxia stress induces immune and metabolic disorders [105]. Recent advances demonstrate crosstalk between adipose, muscle, and bone tissues, emphasizing multi-tissue coordination in growth regulation [106].

Nutritional intervention for metabolic pathway control requires coordinated systems integrating feeding, metabolism, and growth regulation through complex molecular networks [30]. Fasting and refeeding metabolic pathways show intricate patterns affecting growth performance and muscle growth gene expression [107]. Apelin’s physiological response to fasting and refeeding demonstrates neuroendocrine coordination of feed intake and feeding behavior [108]. Starvation and refeeding effects on hybrid grouper encompass growth, gut microbiota, and non-specific immunity through interconnected biological mechanisms [109]. Plasma metabolite mobilization during fasting–refeeding cycles reveals stored reserve utilization strategies [110]. Compensatory growth management requires particular approaches addressing associated fitness costs [111].

### 3.2. TGF-β/Myostatin Signaling

#### 3.2.1. Myostatin Structure, Function, and Growth Inhibition Mechanisms

Myostatin (MSTN) functions as the primary negative regulator of muscle growth through activation of signaling pathways that inhibit myoblast proliferation and differentiation [9,10]. Myostatin inhibition strategies across animal species reveal conserved mechanisms [10]. In Japanese flounder skeletal muscle cells, Myostatin-1 inhibits cell proliferation by suppressing mTOR signaling and myogenic regulatory factors while activating the ubiquitin-proteasomal system [112]. Evolutionary characteristics, biochemical structure, and functional aspects of the *mstn* gene demonstrate conservation across vertebrates [113].

Molecular mechanisms of myostatin action involve binding to activin receptors, subsequent Smad protein activation, and transcriptional changes that inhibit myoblast proliferation and differentiation. Myostatin-1 suppresses cell proliferation through coordinated inhibition of mTOR signaling pathways and myogenic regulatory factors alongside activation of the ubiquitin-proteasomal system in skeletal muscle cells [114]. Myostatin regulation of skeletal muscle growth and development involves multiple cellular pathways coordinating muscle fiber development [9]. Key genes regulating skeletal muscle development and growth in farm animals provide comparative insights applicable to aquaculture species [115]. Understanding fish muscle growth regulation to optimize aquaculture production requires comprehensive knowledge of molecular mechanisms regulating muscle plasticity [116,117]. The growth hormone-insulin-like growth factor system regulates fish skeletal muscle growth through activation of multiple coordinated anabolic pathways [7].

#### 3.2.2. Species-Specific Myostatin Effects and Quantitative Growth Improvements

CRISPR-mediated *mstn* knockout in common carp produces substantial muscle growth enhancement without adverse physiological effects [53]. Transcriptional knockdown improves muscle quality in Nile tilapia through enhanced protein synthesis and reduced protein degradation [54]. Myostatin b functions as a key regulator of somatic growth in interspecific fish hybrids, indicating species-specific regulatory mechanisms [114]. Transcriptome analysis identifies regulatory pathways in fast-growing male tilapia skeletal muscle, revealing myostatin’s role in growth regulation [118].

Myostatin knockout studies demonstrate enhanced muscle mass alongside improved growth rate and feed conversion efficiency across fish species. Myostatin responses show species-specific variations reflecting different genomic arrangements, developmental sequences, and regulatory network architectures. In polyploid species, myostatin regulatory mechanisms become particularly complex due to multiple *mstn* gene copies and sub-genome-specific expression patterns. These findings establish myostatin as a primary target for genetic enhancement strategies that increase fish muscle growth in aquaculture while preserving physiological function and environmental tolerance.

#### 3.2.3. TGF-β Superfamily Integration and Regulatory Networks

TGF-β superfamily signaling controls muscle development through Smad protein-mediated activation of transcriptional networks regulating cellular responses [119]. Common carp contains four Smad4 types, demonstrating how teleost fish developed neo-functionalized pathways through whole-genome duplication events [56]. TGF-β1 suppresses T-cell responses through Smad3- and Foxp3-mediated networks, illustrating immune system integration [55].

Evolutionary timelines and genomic structures of TGF-β-related proteins in Nile tilapia reveal phylogenetic relationships across teleost species [120]. Integration of TGF-β signaling with other growth regulatory pathways generates complex control systems balancing muscle growth with immune function, reproductive development, and environmental stress responses. Anti-Müllerian hormone influences reproductive traits through TGF-Beta pathway activation, demonstrating growth-reproduction coordination [121].

Molecular mechanisms regulating muscle plasticity demonstrate integration across multiple signaling pathways [116]. Tissue-specific coordination between adipose, muscle, and bone tissues reveals that muscle development requires system-wide integration rather than isolated processes [106]. Understanding these integrated networks enables development of comprehensive growth optimization strategies maintaining overall physiological equilibrium across entire biological systems.

#### 3.2.4. Genome Editing Applications and Breeding Strategies

Myostatin genome editing technology demonstrates substantial potential through CRISPR-Cas9 *mstn* knockout producing increased muscle growth across fish species without adverse physiological effects. CRISPR-Cas9 enables farm animal genetic enhancement through myostatin pathway modifications applicable to aquaculture species [122]. Genetic improvement in edible fish shows current status, future prospects, and challenges for CRISPR-based genome engineering [27].

Optimizing commercial traits in aquaculture through gene editing requires techniques accelerating genetic improvement via targeted modifications [123]. Genome editing applications in aquaculture genetic improvement enable superior production outcomes [24]. CRISPR/Cas9 technology modifies *mstn* genes in fish species through precise genetic alterations [124]. CRISPR/Cas genome editing technology offers advantages for fisheries operations through precise trait modifications [25]. Genome editing in cultured fishes provides comprehensive analysis of applications and possibilities [26]. Target gene identification for desirable phenotypes demonstrates genome editing applications in Cyprinidae and Salmonidae species [125].

#### 3.2.5. Environmental and Nutritional Elements Which Affect Myostatin Expression Levels

Understanding TGF-β/myostatin signaling pathways enables development of nutritional approaches and management techniques enhancing growth performance through non-genetic interventions. Myostatin expression manipulation through breeding practices and selection methods utilizes natural genetic variation in myostatin pathway components to improve growth outcomes via conventional breeding techniques. Dietary koumine affects growth performance, intestinal morphology, microbiota, and transcriptional responses, indicating nutritional regulation of growth regulatory networks [126]. Combined koumine and gelsemine effects on growth performance, intestinal health, and transcriptome reveal complex phytochemical interactions potentially affecting myostatin regulation [127].

Genetic improvement and genomic resources of important cyprinid species provide status and future perspectives for sustainable production integrating conventional and molecular approaches [35]. Genome manipulation progress in selected aquaculture organisms expands available tools for myostatin-targeted enhancements [128]. Myostatin represents a key target for genetic modification enhancing economic traits in aquaculture fish species [129].

#### 3.2.6. Implementation Considerations and Future Directions

Implementing myostatin-targeted genetic enhancements requires thorough evaluation of regulatory frameworks, market viability, and public acceptance of genetic modifications. Genome functional annotation advances fish breeding programs through comprehensive genetic characterization [130]. DNA analysis provides advanced methods tracking genetic authenticity and modifications through species identification and targeted genetic alterations [131].

Integrating myostatin research with breeding objectives enables development of comprehensive improvement programs enhancing multiple production traits simultaneously. Biotechnology in fish breeding enables marker-assisted selection and genetic modification for complete systems fulfilling diverse aquaculture improvement needs [132]. Aquaculture production depends on nutrition and selective breeding, requiring integrated approaches uniting genetic improvement with nutritional enhancement [133]. Sustainable management of aquaculture genetic resources needs methods achieving productivity growth while safeguarding environmental health and preserving genetic diversity [34].

### 3.3. Epigenetic Control Mechanisms

#### 3.3.1. DNA Methylation in Growth Regulation and Genomic Architecture

DNA methylation functions as the primary epigenetic mechanism regulating growth-related gene expression through sequence-independent transcriptional modifications [14]. Methylation-based control in aquaculture demonstrates critical roles in regulating growth, reproduction, disease resistance, and stress responses through coordinated genetic and epigenetic mechanisms [14]. Epigenetic modifications function as rapid response systems enabling phenotypic flexibility while maintaining DNA stability, though transgenerational inheritance stability and magnitude require further investigation.

DNA methylation analysis of polyploid fish growth trait heterosis demonstrates that allotriploid growth advantages emerge from coordinated DNA methylation and miRNA regulation [15]. DNA methylation patterns enable coordinated expression of duplicated gene copies, with methylation-dependent sub-genome dominance contributing to hybrid vigor and improved growth performance. DNA methylation and sub-genome dominance control lipid metabolism regulation, resulting in heterosis effects [134]. Methylation pattern stability across generations and sustained heterosis contributions warrant continued investigation.

Allopolyploidy establishment in cyprinid fish requires symmetric sub-genomes and stable homoeolog expression through coordinated epigenetic mechanisms [32]. Parallel sub-genome structure and divergent expression evolution in allotetraploid common carp and goldfish involve DNA methylation regulation [20]. Rediploidization demonstrates genomic diversity emergence from unbalanced to balanced states through epigenetic control of sub-genomic evolution [135]. Common carp domestication involves genetic and epigenetic mechanisms through parallel and asymmetric sub-genome selection [136]. Sub-genomic divergence research studies species development and allopolyploid success rates dependent on epigenetic regulation [137].

DNA methylation patterns show environmental sensitivity enabling rapid adaptation to environmental changes, though inheritance extent and reversibility require continued study. Phenotypic changes and DNA methylation of in vitro aging sperm in common carp demonstrate temporal dynamics of epigenetic regulation throughout development [138]. Understanding how environmental stressors including thermal stress, nutritional status, and pollutant exposure affect gene expression changes leading to population adaptation through long-term genetic modifications requires additional research.

#### 3.3.2. MicroRNA Networks and Post-Transcriptional Control

MicroRNA networks function as complex post-transcriptional control systems precisely managing growth-related gene expression through specific mRNA binding. Teleost microRNA numbers expanded after whole-genome duplication as duplicated protein-coding genes acquired new regulatory elements [13]. Evolution after whole-genome duplication reveals how teleost microRNAs evolved complex regulatory networks [13].

Environmental stress-responsive miRNAs enable rapid management of multiple growth pathways simultaneously [139]. Branched-chain amino acid roles in muscle growth regulation emerge from transcriptome and microRNA sequencing, revealing complex regulatory integration [100]. MicroRNA expression functions as environmental stress response regulator, producing distinct toxicity signatures in organisms experiencing various environmental challenges including pollutant exposure, thermal stress, and nutritional challenges [75].

Profiling miRNAs of teleost fish in response to environmental stress provides comprehensive understanding of stress-responsive regulatory mechanisms [139]. Circulating microRNA tissue origins reveal systemic regulatory functions enabling tissue-to-tissue communication coordinating growth responses. Rainbow trout circulating microRNAs originate from body tissues, with expression pattern synchronization occurring during nutritional or environmental stress [140].

MiRNAs, lncRNAs, and circRNAs between ovaries and testes in common carp reveal sex-specific regulatory networks coordinating growth through reproductive development [103]. Comparative microRNA expression profiles during embryonic development reveal developmental stage-specific regulatory mechanisms [102]. Standard laboratory tests and circulating microRNAs demonstrate immune system responses to paraquat exposure through regulatory mechanisms [141].

#### 3.3.3. Genomic Architecture and Epigenetic Coordination in Polyploid Species

Common carp genome sequence and genetic diversity provide foundational understanding for epigenetic studies [19]. Future genomics and genetic improvement research in allotetraploid common carp will emphasize epigenetic aspects [31]. Genome-wide analysis examining growth patterns in fast-growing common carp strains at two developmental stages demonstrates genetic and epigenetic factor coordination in growth regulation [142]. Chromosome-level genome assemblies providing diploid progenitor-like reference genomes enable understanding of allotetraploid common carp epigenetic architecture [143].

#### 3.3.4. Stability, Heritability, and Mechanistic Understanding

Implementing epigenetic modifications for breeding requires stable, heritable characteristics. DNA methylation patterns survive cell division cycles, though transgenerational transmission mechanisms remain incompletely understood. Environmental factors trigger epigenetic changes in fish persisting across one to multiple generations, but transgenerational inheritance mechanisms and evolutionary adaptation contributions require further study.

DNA hydroxymethylation studies in domesticated species reveal that environmental factors create enduring epigenetic changes enhancing growth, though long-term stability requires additional research [144]. DNA hydroxymethylation and improved growth during Nile tilapia domestication suggest epigenetic contributions to selective breeding success [144]. Epigenetic modifications in aquatic animal hybrids and polyploids reveal intricate inheritance mechanisms involving DNA methylation and multiple epigenetic alterations [145].

Environmental effects during gonadal development demonstrate epigenetic roles in adaptive responses [71]. Gonadal miRNome, transcriptome, and metabolome show intricate molecular reactions to environmental changes [72]. Epigenetic systems function as adaptable control mechanisms helping organisms adapt to environmental challenges, though differentiating between short-term responses and long-term heritable modifications presents ongoing challenges.

#### 3.3.5. Practical Applications in Aquaculture Breeding

Practical implementation of epigenetic mechanisms for aquaculture enhancement through environmental management requires complete validation demonstrating effectiveness. Understanding DNA methylation responses to environmental changes will enable development of management strategies enhancing production efficiency through optimized epigenetic regulation once modification stability and predictability are established.

Nutritional epigenetics research demonstrates that dietary interventions modify regulatory systems through environmental factors controlling growth patterns. Tissue explant studies demonstrate how nutritional signals affect epigenetic changes in the GH-IGF-I axis through direct growth hormone regulatory network modifications [146]. Dietary koumine influences growth outcomes, intestinal structure, microbial composition, and gene expression patterns by modifying epigenetic elements [126]. Combined koumine and gelsemine effects on growth performance, intestinal health, and transcriptome reveal complex epigenetic interactions [127]. Establishing direct causal links between dietary interventions, epigenetic modifications, and long-term growth benefits requires controlled long-term studies.

#### 3.3.6. Integration with Breeding Programs and Future Directions

Implementing epigenetic markers in breeding program design for sustainable genetic resource management requires evaluation of how epigenetic and genetic factors influence trait heritability [34]. Genomic applications for aquaculture genetic improvement require knowledge of epigenetic element influences on observable traits [21]. Evaluating genomic selection potential for aquaculture species breeding improvement requires assessment of epigenetic markers alongside traditional genetic markers [22].

Implementing environmental management strategies regulating epigenetic processes enables cost-effective growth performance improvement through husbandry practices generating beneficial epigenetic modifications, though requiring evidence of environmental factor-epigenetic state-production outcome connections [146]. Research into environmental factors modifying DNA methylation and miRNA expression will yield improved production techniques maximizing growth potential through coordinated genetic and epigenetic regulation [14,15]. Evaluating epigenetic factors is essential for maximizing aquaculture production through understanding nutrition and selective breeding significance [133].

Future research priorities include: (1) determining stability of environmentally triggered epigenetic changes across multiple generations [144,145]; (2) developing rapid screening methods for epigenetic markers in breeding programs [21]; (3) quantifying genetic versus epigenetic contributions to economically valuable traits [14]; (4) identifying environmental factors consistently generating advantageous epigenetic patterns [71,146]; and (5) proving epigenetic intervention effectiveness in commercial aquaculture operations [34]. Evaluating epigenetic approaches for aquaculture improvement depends on addressing current knowledge gaps.

## 4. Genetic Architecture and Genomic Applications

### 4.1. Polyploid Genome Complexity

#### 4.1.1. Evolutionary Legacy of Whole-Genome Duplication

The teleost-specific whole-genome duplication (WGD) occurring 320–350 million years ago fundamentally shaped genetic architecture in commercially important aquaculture species, creating opportunities for sub-functionalization, neofunctionalization, and enhanced regulatory complexity [16]. This ancient polyploidization profoundly impacted molecular networks controlling skeletal muscle growth and organismal development [18].

Rediploidization following teleost WGD represents an ongoing process with heterogeneous rates across genomic regions, creating mosaic architecture where some regions retain duplicated states while others return to single-copy configurations [17]. Genomic reorganization following whole-genome duplication proceeds at variable rates across chromosomal regions, with some segments maintaining tetrasomic inheritance patterns while others revert to disomic patterns through differential gene loss and chromosomal rearrangements. This heterogeneous rediploidization creates complex genomic landscapes requiring specialized analytical approaches for genetic mapping and breeding applications.

Contemporary polyploid species continue demonstrating WGD effects on growth regulation. Distant hybridization and gynogenesis create new genetic improvement opportunities while presenting breeding program challenges [147]. Duplicated genomes undergo complex sub-genome differentiation, expression divergence, and functional specialization, with allotetraploid common carp and goldfish demonstrating how duplicated segments acquire distinct regulatory properties over evolutionary time [20].

#### 4.1.2. Comparative Genomic Architecture Across Polyploid Species

Common carp serves as an exemplary polyploid model system with complex allotetraploid structure containing approximately 50,000 genes across four sub-genomes [19]. Initial genomic insights from BAC-end sequence analysis revealed structural complexity [148], while chromosome-level genome assemblies now provide diploid progenitor-like reference genomes enabling comparative studies [143].

Symmetric sub-genomes and balanced homoeolog expression stabilize allopolyploidy through sophisticated epigenetic regulatory systems coordinating expression between homoeologous copies [32]. Common carp domestication involves asymmetric and parallel sub-genome selection, with different selective pressures acting on distinct sub-genomic regions, creating opportunities for accelerated evolutionary change in economically important traits [136]. DNA methylation and sub-genome dominance control expression balance and contribute to heterosis effects, providing rapid response mechanisms adjusting sub-genomic contributions without genetic mutations [134]. Sub-genomic divergence patterns provide insights into speciation processes and allopolyploid success mechanisms [137]. Integration of high-resolution genomic mapping with phenotypic analysis demonstrates that growth heterosis involves complex interactions between sub-genomic contributions, epigenetic regulation, and environmental factors [15].

#### 4.1.3. Comparative Analysis: Cyprinids and Other Polyploid Taxa

While common carp represents the most extensively studied polyploid aquaculture species, comparative analysis across cyprinid species reveals both conserved mechanisms and lineage-specific adaptations. Goldfish (*Carassius auratus*) shares the tetraploid state with common carp but exhibits distinct patterns of sub-genome evolution and expression divergence [20]. Crucian carp demonstrates unique regulatory patterns in triploid forms, with elevated GH/IGF axis gene expression contributing to growth heterosis [58,59].

Beyond cyprinids, salmonids represent an independent polyploidization event occurring approximately 80–100 million years ago, providing comparative insights into how different lineages manage duplicated genomes. Salmonids retain more duplicated genes than cyprinids at equivalent post-WGD timepoints, suggesting different rediploidization trajectories and selective pressures. This comparative framework reveals that polyploidy consequences depend on both time since duplication and lineage-specific ecological and developmental factors.

Key differences between cyprinid and salmonid polyploidy include: (1) timing of WGD events creating different degrees of genome stabilization; (2) environmental adaptation strategies, with cyprinids showing enhanced tolerance to variable freshwater conditions; (3) growth regulatory complexity, with both groups maintaining expanded growth hormone gene families but different sub-functionalization patterns; and (4) breeding system implications, with cyprinids generally showing more stable tetraploid inheritance compared to residual tetrasomic inheritance in some salmonids.

#### 4.1.4. Functional Implications of Polyploidy for Growth Regulation

Polyploid genome architecture creates both opportunities and challenges for growth optimization in aquaculture [16,31]. The retention of multiple gene copies enables: (1) dosage-dependent effects increasing baseline expression levels [20]; (2) sub-functionalization allowing tissue-specific or developmental stage-specific specialization [18,20]; (3) neofunctionalization creating novel regulatory mechanisms [16,17]; and (4) buffering against deleterious mutations through genetic redundancy [32].

Polyploidy also presents challenges including: (1) increased regulatory complexity requiring coordinated control of multiple homoeologs; [32,136] (2) potential for dosage imbalance affecting stoichiometric protein complexes [31]; (3) reduced efficiency of selection against slightly deleterious alleles due to masking effects [23]; and (4) technical complications for genetic mapping and marker-assisted selection due to complex segregation patterns [149,150].

Genomic approaches in allotetraploid common carp demonstrate expanding potential while acknowledging these inherent challenges [31]. Genetic improvement and genomic resources of important cyprinid species provide frameworks for sustainable production [35]. Genome-wide analysis in fast-growing common carp strains reveals temporal genetic architecture and demonstrates practical application of genomic understanding [142].

#### 4.1.5. Genomic Resources Supporting Polyploid Research

Development of comprehensive genomic resources has been essential for understanding and exploiting polyploid complexity in aquaculture species [19]. Beyond reference genome sequences, critical resources include functional annotation databases, expression atlases, and comparative genomic tools [130]. Studies examining complement gene families demonstrate immune system complexity evolution in polyploids, revealing how duplicated immune genes acquired specialized functions following whole-genome duplication [151]. Comparative analysis of hypoxia response elements identifies functional regulatory sequences across duplicated genomic regions, showing how regulatory element duplication contributes to environmental adaptation [152]. Transcriptome analysis under hypoxia stress reveals coordinated responses across duplicated pathways, demonstrating functional integration of paralogs in stress responses [153].

These genomic resources provide foundational infrastructure for advanced breeding applications and functional genomic studies [21,31]. Integration of structural genomics, transcriptomics, and epigenomics enables comprehensive understanding of how polyploid genomes regulate complex traits like growth performance [130,142]. Importantly, resources developed for common carp serve as models for other polyploid aquaculture species, accelerating genomic research across taxonomically diverse groups [35].

#### 4.1.6. Implications for Breeding and Selection

Understanding polyploid genome complexity has direct implications for genetic improvement strategies. Traditional quantitative genetics approaches must be modified to account for polysomic inheritance patterns and the presence of multiple functional alleles per locus [23,31]. Genomic selection offers advantages in polyploid species by capturing effects across all homoeologs simultaneously, though marker density requirements are substantially higher than in diploid species [22,149].

The presence of sub-genome-specific variants creates opportunities for targeted selection on particular sub-genomes that contribute disproportionately to economically important traits [20,136]. However, this also requires careful attention to maintaining balanced sub-genomic contributions to avoid disrupting established regulatory networks [32]. Future breeding strategies should integrate understanding of sub-genome dynamics, epigenetic regulation, and environmental responsiveness to maximize genetic gain while preserving the adaptive flexibility that polyploidy provides [21,31,34].

### 4.2. Molecular Breeding Technologies

#### 4.2.1. Genomic Mapping and Quantitative Trait Analysis

High-density genetic linkage maps enable precise localization of genomic regions controlling economically important traits. Fine mapping demonstrates practical utility for identifying genes underlying feed conversion efficiency in common carp [150]. QTL mapping reveals temporal specificity of genetic effects, with overwintering studies in Songpu mirror carp showing stage-specific genomic influences that require comprehensive developmental approaches for effective breeding program design [154].

Association studies between genetic variants and growth indicators provide mechanistic insights. GH-1 polymorphisms show context-dependent effects across ecosystems [39], while multiple gene expression studies reveal polygenic growth regulation [42]. Growth hormone gene polymorphisms demonstrate significant associations with growth traits in Caspian Sea populations [41]. Novel diplotypes in *GH* genes and insertion/deletion polymorphisms in *IGF-I* regulatory regions provide additional marker candidates for growth trait selection [43,154].

Genome-wide association studies (GWAS) enable comprehensive surveys of genomic variation, revealing highly polygenic growth trait architecture in aquaculture species [23]. SNP arrays provide important technological advancement for cost-effective large-scale genotyping, enabling GWAS feasibility in non-model species and discovery of small-to-moderate effect variants [155]. GWAS applications demonstrate that fish growth traits involve numerous distributed genomic loci with individual loci explaining small phenotypic variance proportions, necessitating genome-wide rather than single-gene approaches. Comparative genomic analyses reveal selection signatures through whole-genome resequencing in genetically selected populations, providing insights into evolutionary processes underlying genetic improvement programs [156].

#### 4.2.2. Genomic Selection Implementation and Optimization

Genomic prediction enables genetic merit estimation for complex traits using genome-wide markers, potentially accelerating improvement compared to phenotype-based selection. Current genomic selection applications demonstrate broad applicability across diverse fish taxa, offering advantages for expensive or difficult-to-measure traits like disease resistance and feed efficiency [22].

Low-density marker panels achieve comparable prediction accuracies to high-density genotyping at reduced costs, making genomic selection accessible for resource-limited breeding programs [149]. Applications in aquaculture species demonstrate performance across species, traits, and genotyping platforms. Advanced statistical methods account for population structure, environmental effects, and genotype-by-environment interactions. Targeted SNP approaches using genome-wide association analysis results optimize prediction accuracy–cost trade-offs for disease resistance traits [157,158].

#### 4.2.3. Marker-Assisted Selection: Current Applications and Limitations

Marker-assisted selection (MAS) utilizing growth-related genetic markers represents practical implementation of molecular breeding technologies with demonstrated applications across livestock species including fish [159,160]. Association studies between growth hormone gene polymorphisms and growth traits demonstrate practical marker applications, with GH-1 variants showing significant associations with growth indicators across different ecosystems [39]. Novel diplotypes in the *GH* gene associate with body weight traits around the first overwintering period, providing validated markers for selective breeding programs [40]. Single-nucleotide polymorphism arrays provide cost-effective large-scale genotyping platforms enabling marker-assisted selection implementation across aquaculture species [155].

#### 4.2.4. CRISPR-Cas9 Genome Editing: Applications and Precision

Genome editing represents transformative technology for aquaculture improvement through precise genetic modifications [25,26]. Targeted knockout of the *mstn* gene in common carp demonstrates successful application for growth enhancement, with myostatin deletion resulting in improved muscle development and production performance while maintaining low off-target effects [53]. Transcriptional knockdown of *mstn* encoding myostatin improves muscle quality in Nile tilapia through enhanced protein synthesis, illustrating broad applicability across commercially important species [54].

CRISPR/Cas9 technology enables enhancement of desirable traits including growth improvement, disease resistance, and environmental adaptation in aquaculture species [124]. Opportunities for application in farm animal genetic improvement demonstrate broad potential, with aquaculture benefiting from developments in terrestrial animal breeding [122]. Strategies for optimizing commercial traits through gene editing provide approaches for accelerating genetic improvement through targeted modifications of growth-related pathways [123]. Advances in genome manipulation across selected aquaculture organisms provide comprehensive overview of editing technologies [128]. Identification of genes suitable for editing to improve economic traits in aquaculture fish species establishes specific targets for genetic modification [129].

#### 4.2.5. Global Regulatory Frameworks and Implementation Challenges

Regulatory landscapes for genome-edited aquaculture products vary substantially across global regions, creating barriers to international technology transfer and commercial deployment [25,26]. Regulatory frameworks differ in how they classify gene-edited organisms relative to transgenic organisms, with some jurisdictions applying stringent GMO regulations to all genome-edited products regardless of foreign DNA presence, while others distinguish between these categories [24,123].

These regulatory divergences create strategic challenges for breeding programs requiring: (1) region-specific product development strategies; (2) comprehensive documentation systems for regulatory compliance across jurisdictions; (3) consumer acceptance research in target markets; (4) intellectual property strategies accounting for regional differences; and (5) long-term monitoring systems for environmental and food safety assessment [27,34]. Genome functional annotation advances support breeding programs while facilitating regulatory submissions through comprehensive genetic characterization [130]. DNA analysis methods enable species identification and genetic modification tracking, providing essential tools for regulatory compliance and product authentication [131].

#### 4.2.6. Integrated Technology Deployment

Integration of multiple technologies enables comprehensive improvement strategies combining genomic selection with marker-assisted selection, gene editing, and reproductive technologies to maximize breeding efficiency [21,34]. Biotechnological innovation in fish breeding encompasses opportunities ranging from marker-assisted selection through genetic modification, creating comprehensive toolkits for addressing diverse aquaculture improvement objectives [132].

Optimal breeding strategies often employ technologies sequentially: (1) genomic selection for polygenic trait improvement such as growth rate and feed efficiency [22,23]; (2) marker-assisted selection for major-effect loci including disease resistance alleles [159,160]; (3) genome editing for targeted modifications of specific pathways such as *mstn* knockout [24,123]; and (4) epigenetic management for environmental responsiveness [14,145]. This sequential approach maximizes genetic gain while managing implementation costs and regulatory considerations (Table 2).

Alternative strategies employ technologies in parallel, implementing simultaneous genomic selection across multiple traits while developing gene-edited lines for specific improvements. This approach accelerates overall genetic gain but requires substantial resource investment and careful genetic diversity management to avoid inbreeding depression [21,34].

#### 4.2.7. Economic Impacts and Implementation Pathways

Economic impacts of genetic improvement technologies reveal substantial returns on investment through enhanced production efficiency, reduced production costs, and improved product quality [4]. Genetically improved common carp stock demonstrates significant effects on pond ecosystem productivity with implications for selective breeding in nature-close conditions [33]. Regional aquaculture status, challenges, and trends illustrate implementation opportunities and constraints [5]. Integration of nutrition and selective breeding in aquaculture production emphasizes combined approaches optimizing both genetic and nutritional factors [133].

Implementation pathways require: (1) technology transfer mechanisms from research institutions to commercial operations [21]; (2) training programs for technical personnel [22]; (3) infrastructure development for genotyping and data management [130]; (4) regulatory navigation support for novel technologies [27]; and (5) market development strategies addressing consumer acceptance [34]. Successful implementation depends on coordinated efforts across research, industry, regulatory, and extension sectors to translate technological advances into practical production improvements.

Genetic improvement initiatives demonstrate successful implementation of molecular breeding technologies across diverse species and production systems [161]. Selective breeding reviews demonstrate comprehensive breeding approaches [162]. Biotechnological innovation in fish breeding encompasses opportunities ranging from marker-assisted selection through genetic modification, creating comprehensive toolkits for addressing diverse aquaculture improvement objectives [132]. Status, constraints, and prospects on CRISPR-based genome engineering in edible fish demonstrate current capabilities and future directions [27]. Genetically improved aquaculture species demonstrate successful technology implementation in various regional contexts [163]. Genome editing applications in Cyprinidae and Salmonidae species identify suitable target genes for desirable phenotypes [125]. Current research directions and applications emerge from genomics in aquaculture symposia [164].

## 5. Sustainable Integration and Future Directions

### 5.1. Integrated Multi-Trophic Aquaculture Systems

#### 5.1.1. Ecosystem-Based Production and Nutrient Optimization

Integrated multi-trophic aquaculture (IMTA) functions as an ecosystem-based farming system utilizing species from different trophic levels to maximize nutrient utilization while minimizing environmental impacts [165,166]. IMTA systems represent sustainable approaches advancing aquaculture operations through strategic species combinations [167]. The evolution of IMTA design and components enables optimization and diversification, creating pathways toward enhanced sustainability through valorization of waste products [6].

These systems combine finfish with shellfish and seaweed production to achieve maximum resource efficiency through planned food web arrangements [168]. Modular integrated multi-trophic recirculating aquaculture systems (IMTRAS) cultivating fish, mussels, sea cucumbers, and macroalgae demonstrate superior performance and waste removal efficiency through coordinated multi-species interactions [169]. Integration of blue mussels with kelp cultivation results in higher production levels and improved kelp biomass quality [170]. Large-scale seaweed and shellfish aquaculture operations in Europe show multiple impacts on nutrients, carbon cycling, and fisheries when evaluated through operational oceanographic models [171]. Properly managed IMTA systems achieve superior nutrient retention compared to monoculture approaches by optimizing nutrient cycling and minimizing environmental pollution [172].

Freshwater IMTA-aquaponic systems implementation promotes sustainable environmental practices through effective nutrient management, optimized hydraulic loading rates, and improved feed conversion ratios [173]. Novel integrated modeling tools enable optimization of seafood nutritional value and environmental sustainability, facilitating comparative assessments between IMTA and monoculture systems [174].

#### 5.1.2. Genetic Optimization for Integrated Systems

IMTA systems require strategic genetic improvement approaches that enhance individual species performance while preserving beneficial ecological interactions between cultivated organisms [172,174]. Genetic selection programs must optimize species-specific traits while considering system-wide performance requirements, potentially balancing maximum individual species productivity with overall operational stability. Environmental adaptation genetic traits become particularly critical when different species must function under shared environmental conditions [33].

Potential genetic improvement objectives for IMTA systems include: (1) fish strains with optimized feed conversion efficiency to minimize nutrient release [133]; (2) shellfish strains with enhanced filtration capacity [169]; (3) seaweed varieties with superior nutrient uptake efficiency [170,171]; and (4) species with coordinated growth rates enabling synchronized harvest timing. Genetic improvement of each component species requires consideration of impacts on other system components, suggesting breeding objectives should adopt systems-level rather than single-species optimization perspectives [174].

Sustainable management of genetic resources requires comprehensive evaluation of genetic enhancement impacts on ecosystem function, balancing productivity improvement with environmental protection [34]. Genetically improved common carp stocks demonstrate effects on pond ecosystem productivity, illustrating how selective breeding can enhance production efficiency while maintaining environmental sustainability in nature-close conditions [33]. Innovative technologies for fish breeding with minimal environmental impact demonstrate sustainable breeding approaches applicable to integrated systems [175]. Optimizing aquaculture production requires comprehensive approaches integrating nutritional strategies with selective breeding programs [133].

### 5.2. Climate Adaptation and Resilience

#### 5.2.1. Climate-Resilient Strain Development and Multi-Stressor Tolerance

Managing reproductive biocomplexity under climate change requires innovative approaches integrating climate adaptation objectives into genetic improvement strategies [28]. Identifying and enhancing natural genetic variation conferring resilience to projected climate changes—including altered temperature regimes, shifting precipitation patterns, and extreme weather events—represents a critical research priority. Warming waters fundamentally alter fish physiology, requiring adaptive responses through targeted genetic improvement for thermal tolerance [11].

Developing climate-resilient strains through genetic selection requires enhancing heat tolerance, hypoxia resistance, and metabolic flexibility for predicted climate scenarios while preserving genetic diversity for future adaptation capacity. Cellular stress response mechanisms and temperature regulation systems demonstrate conserved evolutionary functions enabling climate adaptation [65]. Genetic pathways underpinning hormonal stress responses in fish exposed to short- and long-term warm temperatures reveal mechanisms supporting climate adaptation strategies [29]. Higher acclimation temperatures affect growth through suppression of *GH* and *IGF* gene expression, activating stress responses that compromise normal growth regulation [66].

#### 5.2.2. Integrated Environmental Stress Responses

Environmental stressor tolerance constitutes a vital component of climate-resilient strain development, with genetic enhancement targeting multiple stress response pathways including thermal stress, hypoxia tolerance, and salinity adaptation. Environmental stressors affecting fish growth operate through complex endocrine control mechanisms [12]. Environmental hypoxia threatens gonadal development and reproduction in bony fishes, requiring adaptive responses [70].

Understanding interactions between various climate stressors enables development of multi-stressor tolerance breeding programs creating comprehensive resilience strategies for performance under multiple simultaneous environmental challenges. Environmental stressor impacts on growth and the GH-IGF1 pathway demonstrate complex stress response mechanisms requiring integrated approaches [176]. Environmental enrichment in fish aquaculture systems serves dual functions improving stress resistance and production outcomes [73].

Fish physiological responses to freshwater salinization at the molecular level become accessible through multi-tissue transcriptomic approaches enabling detailed stress adaptation studies [177]. Environmental factors affecting oxidative status generate physiological responses during environmental stress exposure [178]. Transcriptome analysis reveals molecular underpinnings of hypoxia stress responses through pathways enabling survival under low-oxygen conditions [153]. Genome-wide comparative analysis of hypoxia response elements enables in silico identification of functional regulatory sequences coordinating stress responses [152].

### 5.3. Emerging Technologies and Applications

#### 5.3.1. Advanced Multi-Omics Integration and Precision Phenotyping

Multi-omics approaches reveal molecular factors affecting muscle quality in common carp under different aquaculture management systems through integrated analysis of genomic, transcriptomic, proteomic, and metabolomic data [98]. Comprehensive analytical approaches enable identification of regulatory genes and molecular mechanisms optimizing economically important traits. Metabolomic and transcriptomic analysis of muscle growth provides enhanced understanding of growth mechanisms by demonstrating interactions between metabolic and gene expression pathways influencing growth performance [179].

Environmental effects on gonadal miRNome, transcriptome, and metabolome demonstrate how multi-omics approaches reveal coordinated molecular responses to environmental challenges across growth, reproductive, and metabolic functions [72]. Transcriptomic profiling reveals regulatory genes and molecular mechanisms underlying residual feed intake variation, providing targets for improving feed efficiency [99]. Integrated transcriptome and miRNA sequencing analyses reveal that hypoxia stress induces coordinated immune and metabolic disorders [141]. Transcriptomic analysis provides insights into candidate genes and molecular pathways involved in growth processes across aquaculture species [180].

#### 5.3.2. Precision Aquaculture Technologies

Advanced sensor technologies and real-time monitoring systems enable continuous assessment of environmental parameters and physiological status, supporting data-driven management decisions. Integration of environmental monitoring with genetic information enables precision aquaculture approaches tailoring management practices to specific genotypes and environmental conditions. These technologies facilitate optimization of production systems through adaptive management strategies responding to real-time data on water quality, feeding behavior, and growth performance.

### 5.4. Research Priorities and Implementation Challenges

#### 5.4.1. Critical Knowledge Gaps and Research Needs

Several critical knowledge gaps limit full exploitation of molecular breeding technologies for sustainable aquaculture improvement. Climate-resilient genomic resources require development, including genomic prediction models integrating climate data, identification of multi-stress tolerance genes, and establishment of reference populations spanning diverse thermal environments [11,21,22,28]. Selection approaches must enable prediction of performance under various temperature scenarios while identifying markers for hypoxia tolerance and developing breeding populations maintaining high productivity under elevated temperatures [29,66].

Polyploid-specific breeding methodologies require advancement, including statistical methods accounting for polysomic inheritance, optimization of marker density requirements for polyploid genomic selection, and characterization of sub-genome-specific heterosis contributions [20,23,31,149]. Breeding value estimation systems must achieve high accuracy for tetraploid species while enabling marker reduction approaches that maintain prediction accuracy and facilitate sub-genome-directed selection strategies [32,136].

Integration of epigenetic markers into breeding programs represents an important frontier, requiring quantification of epigenetic variance components for growth traits, development of high-throughput epigenetic screening methods, and validation of transgenerational epigenetic inheritance patterns [14,21,34,145]. Understanding how environmental factors induce heritable epigenetic changes and developing cost-effective epigenetic marker panels that remain stable across multiple generations will enable practical breeding applications [15,143].

IMTA system optimization through genetic approaches requires development of system-level breeding objectives, quantification of genetic correlations between species, and identification of optimal species combinations through genetic modeling [33,34,172,174]. System-specific selection indexes must account for ecological interactions while evaluating productivity improvements at the system rather than individual species level.

#### 5.4.2. Technology Transfer and Capacity Building

Bridging the gap between research advances and practical implementation requires strengthened technology transfer mechanisms [21]. Critical needs include establishing regional genomics hubs providing accessible genotyping services, developing digital platforms for molecular breeding education and training, fostering academic-industry partnerships facilitating knowledge exchange, and building south–south cooperation networks enabling resource and expertise sharing among developing nations [22,34]). Successful genetic improvement initiatives demonstrate the importance of coordinated technology implementation across diverse production systems [161,163].

#### 5.4.3. Integration with Sustainable Development Goals

Molecular breeding technologies must align with broader sustainability objectives, requiring simultaneous advancement of production efficiency, environmental stewardship, and social equity [34]. Policy frameworks should encourage innovation while ensuring environmental protection and genetic resource conservation [33,175]. International collaboration becomes essential for addressing global food security challenges while adapting to climate change impacts through coordinated research, technology sharing, and capacity building initiatives [28].

#### 5.4.4. Balanced Approach to Technological Innovation

Future research must balance technological advancement with practical feasibility, economic viability, and social acceptability [21,27]. While genome editing and advanced genomics offer powerful tools for genetic improvement, successful implementation requires addressing regulatory uncertainty, building public trust, and ensuring equitable access to technologies across diverse production systems and geographic regions [25,34]. Integration of molecular tools with ecosystem-based management approaches enables sustainable aquaculture intensification, where precision genetics supports environmental protection rather than compromising it [133,175].

The molecular revolution in aquaculture genetics provides unprecedented opportunities for sustainable production enhancement. Realizing this potential requires coordinated efforts integrating scientific innovation, practical application, regulatory support, and stakeholder engagement [21]. Success depends on developing technologies that simultaneously address productivity demands, environmental constraints, and climate adaptation needs while maintaining genetic diversity and supporting resilient food production systems [28,34].

## 6. Conclusions

This comprehensive review of 180 peer-reviewed studies reveals that molecular growth regulation in aquaculture operates through integrated multi-level networks—the GH-IGF axis, TGF-β/myostatin signaling, and epigenetic mechanisms—that respond dynamically to environmental and nutritional inputs. Polyploid genome complexity, resulting from ancient whole-genome duplications, creates unique opportunities for genetic improvement through sub-functionalization and neofunctionalization while presenting challenges for breeding program implementation.

Advanced molecular technologies including genomic selection, marker-assisted breeding, and CRISPR-Cas9 genome editing enable precise genetic management with demonstrated growth improvements of 15–30% in target species. However, widespread implementation faces regulatory barriers, economic constraints, and technical complexity that limit adoption, particularly in developing regions. Epigenetic mechanisms provide rapid environmental adaptation pathways complementing traditional genetic approaches, though transgenerational stability requires further validation.

Integration of precision genetics with sustainable production systems—including IMTA and climate-resilient strain development—demonstrates that molecular breeding can simultaneously enhance productivity and environmental stewardship. Future success depends on three critical elements: (1) developing climate-adapted strains through multi-omics integration and polyploid-specific breeding methodologies; (2) validating epigenetic markers for practical breeding applications; and (3) establishing technology transfer mechanisms enabling equitable access across diverse production systems.

The molecular revolution in aquaculture genetics provides unprecedented tools for addressing global food security while adapting to climate change. Realizing this potential requires coordinated international efforts integrating scientific innovation with practical implementation, regulatory support, and stakeholder engagement to build resilient, sustainable aquaculture systems capable of meeting growing protein demands while protecting environmental health.

## Figures and Tables

**Table 2:** Comparison of molecular breeding technologies in aquaculture.

Technology	Mechanism	Advantages	Limitations	Implementation Timeline	Economics Impact
Marker-assisted selection (MAS)	DNA markers linked to QTLs	Proven technology Cost-effective Regulatory approval established Preserves genetic diversity	Limited to known QTLs Moderate genetic gain Requires large populations	Immediate	Low-moderate investment, gradual returns
Genomic selection	Genome-wide SNP prediction	Whole-genome coverage Higher accuracy Suitable for complex traits Accelerated breeding cycles	High initial genotyping costs Requires statistical expertise Population-specific models	2–5 years	Moderate-high investment, accelerated returns
CRISPR/Cas9 genome editing	Targeted gene modification	Precise modifications Large effect sizes Rapid trait improvement Novel trait creation	Regulatory uncertainty Technical complexity Off-target concerns Consumer acceptance issues	5–10 years	High investment, potentially high returns
Epigenetic selection	Environmental modulation	Rapid environmental adaptation Transgenerational effects Low implementation costs Environmentally responsive	Limited understanding Inheritance uncertainty Environmental dependency	3–7 years	Low moderate investment, variable returns
Multi-omics integration	Detailed molecular profiling	Systems-level understanding Predictive modeling Multiple trait optimization Precision breeding	High analytical complexity Substantial data requirements Integration challenges	5–15 years	High investment, long-term returns

Note: Cost estimates and implementation timelines represent industry averages based on published case studies and expert consultation. Actual values vary depending on species, infrastructure availability, and regional regulatory requirements. Economic impact assessments reflect projected returns relative to conventional breeding approaches under optimized implementation scenarios.
